# Studying the syndrome of absenteeism among staff of the capital emergency center in relation to the severity of insomnia

**DOI:** 10.1192/j.eurpsy.2026.12090

**Published:** 2026-07-31

**Authors:** P. Sarantuya, S. Surlegbaatar, A. Altantsetseg, T. Urtnasan

**Affiliations:** 1 Medicine Department, Etugen University; 2 Graduate School оf Education, Graduate University оf Mongolia; 3 Basic Medicine Department, Etugen University, Ulaanbaatar, Mongolia

## Abstract

**Introduction:**

The shift work and irregular work schedules of emergency providers are associated with physical and psychological problems, such as sleep disorders. However, emergency medical workers in Ulaanbaatar work an average of 62 hours more per month, indicating a high workload and low rest. Therefore, cases of quitting work due to workload are increasing. As of 2020, it was estimated that four out of 10 doctors had at least one symptom of burnout syndrome.

**Objectives:**

The aim was to investigate the burnout syndrome among doctors and medical specialists of the Ulaanbaatar emergency medical center in relation to sleep disorders. The objective of the study was to examine the relationship between burnout syndrome and sleep disorders, as well as regression.

**Methods:**

The overall assessment of the study was performed using Fisher’s exact test. Burnout syndrome was assessed using the Maslach Burnout Inventory (MBI), which evaluates three domains of psychological change: emotional exhaustion, reduced passion for work, and diminished personal accomplishment, through a total of 22 items. The Insomnia Severity Index (ISI) was employed as a standardized questionnaire to determine the severity of insomnia.

**Results:**

Emotional exhaustion was weakly positively correlated with the Insomnia Severity Index (r=0.38, p=0.00). Depersonalization was moderately positively correlated with the Insomnia Severity Index (r=0.40, p=0.00). Personal accomplishment was strongly positively correlated with depersonalization (r = 0.84, p=0.00). The number of work shifts per month was inversely correlated with emotional exhaustion (r=-0.22, p=0.00), while it was weakly positively correlated with the length of vacation between work shifts (r=0.23, p=0.00). In simple linear regression analysis, an increase of one in the number of work shifts was associated with a 15.6% decrease in emotional exhaustion and a 21% decrease in depersonalization, which was statistically significant (p = 0.00, p = 0.02). A one-point increase in the Insomnia Severity Index score increased emotional exhaustion by 15.6% and depersonalization by 29.01%, which was statistically significant (p=0.00). However, a one-point increase in emotional exhaustion score increased insomnia severity by 97.92% and depersonalization by 151%, which was statistically significant (p=0.00, p=0.00). A one-point increase in depersonalization score increased emotional exhaustion by 46.77% and insomnia severity by 56.94%, which was statistically significant (p=0.00, p=0.00). In simple logistic regression analysis, a one-point increase in emotional exhaustion score increased insomnia severity by 1.25 times, which was statistically significant (p=0.00).

**Conclusions:**

The severity of insomnia is weakly positively correlated with burnout syndrome. Mood swings affect the severity of insomnia by 1.25 times.

**Disclosure of Interest:**

None Declared

